# Seroprevalence of hepatitis E in swine abattoir workers

**DOI:** 10.4314/ahs.v17i4.9

**Published:** 2017-12

**Authors:** Aquino Qouilazoni Ukuli, Kizito Kahoza Mugimba

**Affiliations:** Makerere University, College of Veterinary Medicine Animal Resources and Biosecurity (COVAB), Department of Biotechnical and Diagnostic Sciences

**Keywords:** Hepatitis E, seroprevalence, swine abattoir workers

## Abstract

**Background:**

Hepatitis E (HE) caused by Hepatitis E virus (HEV) is an emerging global public health threat. It has been identified as potentially zoonotic and swine act as main reservoirs.

**Objectives:**

The objective of this study was to determine the seroprevalence and risk factors associated with HEV in swine abattoir workers.

**Methods:**

This was a cross-sectional study where 45 workers were sampled (N=50), serum collected and tested for presence of anti HEV IgM using ELISA.

**Results:**

A seroprevalence of 13.3% was obtained with the highest 50% among slaughterers and the lowest amongst sanitary cleaner, cloth cleaners and inspector. Those in direct contact with live pigs, their carcasses and tissues were at a higher risk compared to those in indirect contact. Seroprevalence was seen to increase with age, with the highest rate among those above 24 years.

**Conclusion:**

There is silent HE virus infection in abattoir workers at Wambizi as reflected by presence anti HEV IgM in 13% of the tested serum. However, no single case of HE has ever been reported in swine abattoir workers or general population in Kampala city. This silent maintenance of HEV infection amongst swine abattoir workers is an occupational risk that could challenge public health systems.

## Introduction

### Back ground

Hepatitis due to HEV is an emerging global public health problem. Twenty million cases are reported annually of which 3.4 million are acute resulting in 70,000 deaths worldwide^1^. HEV is the second most important cause of acute clinical hepatitis in adults in Asia and Africa^2^. The disease had been considered a sanitation problem in resource limited countries. However, the zoonotic form has emerged in industrialized countries with high sero-prevalence^3^–^6^. Hepatitis E was first documented in Uganda in 2007 as a sporadic outbreak in Kitgum where it caused over 10,196 illnesses and 160 deaths^7^. The primary exposure in this outbreak was not elucidated. The disease has since remained endemic in Karamoja Sub-region with high morbidity. Between 2009 and September 2012, 987 cases of HE were reported with 22 deaths^8^. HEV is mainly transmitted through the oral-fecal route. Consumption of feacal contaminated water and food has been implicated in major outbreaks^9^–^15^. HEV is zoonotically transmitted mainly through consumption of meat from infected animals^16^–^18^. Swine have been singled out as the most important source of zoonotic infection; and HEV seroprevalence of 51% has been reported in pig handlers and 14% in wild boar handlers^19^–^22^. The zoonotic transmission of HEV presents a high risk to a larger population especially for individuals that work in game parks, abattoirs, butcheries and consumers of animal products such as pork, pig liver and undercooked game meat^9^,^23^. Given that many people in Uganda depend on pigs and their products for a livelihood, HEV should be of public health concern.

HEV is a positive sense; non enveloped single stranded RNA virus of the family Hepeviridae^24^–^26^. It causes viral hepatitis in humans that has been reported worldwide since it was first reported in Burma, India in 1955^27^; and confirmed in 1983^28^. The disease affects mainly persons aged 15–40 which is the most productive age bracket. Severity of HE increases in pregnant women, with a mortality rate as high as 25%. The disease poses economic challenges not only as loss of lives and huge medical bills but also millions of man hours are lost due to direct or indirect disease devastation. Although the zoonotic form has been suggested as the cause of the disease in the developed world^4^–^9^–^18^–^29^–^31^, zoonotic trasmission is not documented in Uganda.

There are five HEV genotypes .i.e.. HEV I, II, III, IV reported to affect humans and the fifth genotype causes disease in avian species^32^,^33^. However, only genotypes III and IV infect both swine and humans; and are responsible for the zoonotic HEV infections^34^. This underscores the role swine and swine handlers could play in the epidemiology of HEV that has unfortunately not been studied in Uganda. Swine HEV (genotypes III and IV) shares similar antigenic epitopes with the capsid protein of the human HEV, with the accompanying antibody cross reactivity and 90–100% sequence homology^17^–^23^–^35^–^39^, a clear indication of a common ancestor. Such findings coupled with the popularity of pigs as a source of food and icome in Uganda underscore the potential role of swine to the epidemiology of HE. Bearing in mind that abattoir workers are in constant interaction with pigs and their products, the possibility of introducing the virus in their communities cannot be underestimated. Furthermore, research has shown that HEV can also be transmitted through blood transfusion^40^. This study sought to understand the seroprevalence of HE and the factors associated with transmission in swine abattoir workers in Kampala. The findings offer evidence based guidelines to public health practitioners and epidemiologists in understanding HEV dynamics.

## Methology

A cross-sectional study was carried out in Wambizi pig abattoir located in Nalukolongo, Lubaga Division, in Kampala, Central Uganda. The abattoir supplies pork to consumers in and around Kampala. The study population involved abattoir workers at the different levels of the pork value chain within and around the abattoir. Although sampling was intended for all the 50 workers, only forty five workers were present at the day of sampling. Blood (2mls) was collected from 45 abattoir workers (90%) by venous puncture into plain vacutainer tubes labeled with the subjects' unique identification number. Quantitative data was collected using a structured questionnaire to assess the risk factors for transmission of HEV. This Sampling was conducted in April 2015. Ethical clearance to conduct this study was obtained from Makerere University, School of Medicine's research and ethics committee. Informed consent was sought from the participants before sampling.

In the laboratory, the samples were centrifuged at 3000 rpm for 10 minutes and the supernatant (serum) was recovered and stored at -20°C until analysed. Analysis was done using DS-EIA-Anti HEV-M ELISA IgM assay kit (DSI S.r.l, VA, Italy) for HEV IgM antibodies according to the manufacturer's instructions. Results from the structured questionnaire were analysed using STATA statistical package version 1.2 (College station, TX: StataCorp LP.) to determine the degree of association between risk factors and the results.

## Results

Six out of forty five samples collected tested positive for anti HEV IgM, giving a general seroprevalence of 13.3% at a confidence interval of 95%. Specifically seroprevalence varied with the activity someone was involved in (identified as risk factors) as summarised in table 1.

**Table 1:** Analysis of factors associated with HEV in workers at Wambizi pig abattoir.

	Hepatitis E		N=45				
Variable	Neg N=39	Pos N=6	n	cPR*	aPR	95%CI	*p*-value
**Occupation** Cloth cleaners Sanitary Cleaner Supplier Cook Trader Slaughter Inspector(reference)	1(100%) 1(100%) 1(50%) 3(75%) 8(88.9%) 24(88.9%) 1(100%)	0(0%) 0(0%) 1(50%) 1(25%) 1(11.1%) 3(11.1%) 0(0%)	1 1 2 4 9 27 1	1.0 1.0 5.0×10^6^ 2.5×10^6^ 1.1×10^6^ 1.1×10^6^ 1.0	0.7 1 4.2e6 2.1e6 9.6e5 8.8e5	0.04;14.09 0.68;14.65 3.1e5;8e7 1.9e5;2.4e7 4.9e4;1.9e7 1.02e5;7.6e6	1.0 1.0 **<0.001** **<0.001** **<0.001** **<0.001**
**Work duration** <4yrs >4yrs (reference)	23(95.8%) 16(76.2)	1(4.2%) 5(23.8)	24 21	1.05 1.0			0.34
**Sex** Females Males (reference)	12(85.7%) 27(87.1%)	2(14.2%) 4(12.9%)	14 31	1.107 1.0			0.9
**Age** <24yrs 24–35yrs >35yrs	4(100%) 23(85.2%) 12(85.7%)	0(0%) 4(14.8%) 2(14.3)	4 27 14				
**Eat pork** Yes No	37(88.1%) 2(66.7)	2(18.2%) 4(11.8%)	11 34	1.55 1.0			0.588

* cPR= Crude Prevalence ratio

## HE Seroprevalence in different occupations

The results showed 50% (3/6) of the positive samples were from study subjects directly involved in the slaughter of animals (slaughterers), 16.67% (1/6) for the traders, suppliers and cooks.

## Discussion

This study was done in a population of swine abattoir workers where HE infection has not been reported either in workers or in pigs. A seroprevalence of 13.3% (n=45) was observed. The general seroprevalence range reported in developing countries is 3–80%^41^–^42^. Studies in healthy individuals in the United States where HEV is not endemic indicated a significant proportion of anti HEV antibodies of up to 22%^43^–^44^. This increased seroprevalence in a developed country is associated with consumption of swine products such as pork and pig liver and travelling to areas where HEV is endemic. Other studies done in Africa indicated a seroprevalence ranging from 6–80%^41^. In Egypt a country where pork consumption is uncommon due to the dominant Islamic religion, HEV seroprevalence was reported to be 80%^41^. This can be attributed to other routes of transmission r than pork consumption probably the oral faecal route or other zoonotic sources. Hepatitis E virus RNA has been detected in faecal wastes and has been seen to persist in the environment^45^–^46^. In developing countries where farming methods and sewage treatment are poor, introduction of faecal wastes in the water system is likely. This compromises water quality and increases chances of disease outbreak. Exposure to animal wastes and fluids is another important source of infection^47^.

Increased seroprevalence was observed in people who were in direct contact with pigs, pig wastes and carcasses such as slaughterers, traders and suppliers. This is comparable to previous studies which indicated a seroprevalence as high as 66%^19^,^47^ among swine farmers. This may be due to the fact that these individuals are exposed to swine wastes and blood when cleaning and assisting in birth. Of the 6 positives, 50% was observed in slaughterers (3/6), 16.67% in cooks and 16.7% (1/6) was observed in Traders and suppliers. There was an association between the type of work and HEV seropositivity (Table 1) suggesting animal-to-human transmission probably through animal wastes and other tissues. In this study exposure could also be from water or hands to the food they eat since no relationship was found between pork consumption and HEV seropositivity. On the contrary, inspectors who are equally exposed did not show any sero-reactivity for IgM. This may be due to fact that inspectors have a higher level of education and practice good personal hygiene than other workers, as this is reported to influence HEV exposure^47^. However, it remains a speculation as the association between seropositivity and education level of the participants was never investigated in the current study.

None of the seropositive subjects was showing clinical signs. However, HEV infections are associated with HEV viremia and faecal excretion of the virus for a few weeks sometimes in absence of clinical symptoms^41^. The detection of anti HEV IgM antibodies indicates a recent active infection which suggests that the positive subjects had recently acquired the infection. These findings therefore allude to the fact that HEV is being transmitted asymptomatically among swine abattoir workers but the implication of this is moot. Although pigs have been reported as a source of zoonotic HEV infection in different regions of the world,^19^, there is no documentations to show this in Uganda. There were two suppliers in the tested population of which one was positive. Since suppliers move to several pig farms purchasing animals, it cannot be concluded whether he got infected in the abattoir or the farms where he buys animals. Studies have shown that persons engaged in occupations related to swine farming were found to have a higher risk of infection than those in other occupations^48^.

The results show a high seroprevalence of 23.8% (n=21) among those who have spent over four years as compared to the 4.2% (n=24) among those who have spent 4 years and less in the abattoir. This compares to other studies where period of occupational exposure to swine was associated with HEV infection^19^. IgM antibody levels decline rapidly in 6 weeks^41^ while IgG persists in the host for long period providing protection against subsequent infections. The duration of persistence of circulating IgG antibodies remains unclear as they have been found in healthy subjects living in all geographical areas, although the prevalence varies widely^41^. Since the workers were not tested for IgG antibodies, it cannot be determined whether those who had spent four years and less in the abattoir had already been exposed and were immune-competent or those above four years in the abattoir their immunity had waned.

Seroprevalence of HE was seen to increase with age. These results indicate a seroprevalance of 18.8% and 18.3% among the middle aged workers between 24 and 35 years and above 35 years respectively as compared to none among those below 24 years. This trend has been observed in other studies where a prevalence of 3 to 30% was observed in young adults between ages of 20 and 40 years than in children (0.3 to 10%)^46^. This can be attributed to the fact that middle aged adults may be involved in activities such as tending to animals and casual labour in places where they eat and drink poor quality food and water. However in another study in Nigeria a high seroprevalence was observed among those below 10 and above 60 years and the lowest seroprevalence among those between 20 and 40 years^47^. In this study, the increased seroprevalence among the middle aged may be explained by the fact that most of them are energetic males who are involved in slaughtering animals in the abottoir which increases their degree of exposure.

Males had a high seroprevalence compared to females. This is beacause males are more involved in activities of slaughtering and supplying animals thus more exposed to animals and animal carcasses compared to females.

Pork consumption is advanced as another source of HEV infection^23^–^49^–^51^. The results of this study however indicate no association of HEV infection with consumption of pork (P value= 0.267). Probably, workers eat well cooked pork and other vehicles of HEV transmission such as exposure to the animal wastes during slaughtering and handling come into play. Although the animals were not tested the results indicate that those in direct contact with animals have higher chances of acquiring the infection as compared to those who were in indirect contact within the abattoir environment.

## Conclusion

There is silent HEV infection amongst abattoir workers at Wambizi, as reflected by the high seroprevalence and this should raise public health concern. This silent infection is maintained by other means than pork consumption. Direct contact with pigs and pig products has high association with HEV infection. More investigation involving pigs and more humans in various occupations should be conducted to help understand the epidemiology of HEV and guide control measures. Bearing in mind that recent HE outbreaks in Uganda have been sporadic with an elusive primary source, such studies would improve our understanding of HEV epidemiology.
